# Poly-ε-lysine based hydrogels as synthetic substrates for the expansion of corneal endothelial cells for transplantation

**DOI:** 10.1007/s10856-019-6303-1

**Published:** 2019-09-04

**Authors:** Stephnie Kennedy, Rebecca Lace, Constandinos Carserides, Andrew G. Gallagher, Donald A. Wellings, Rachel L. Williams, Hannah J. Levis

**Affiliations:** 1 Department of Eye and Vision Science, Institute of Ageing and Chronic Disease, University of Liverpool, 6 West Derby Street, Liverpool, L7 8TX UK; 2 SpheriTech Ltd, Business and Technical Park, The Heath, Runcorn WA7 4QX UK

## Abstract

Dysfunction of the corneal endothelium (CE) resulting from progressive cell loss leads to corneal oedema and significant visual impairment. Current treatments rely upon donor allogeneic tissue to replace the damaged CE. A donor cornea shortage necessitates the development of biomaterials, enabling in vitro expansion of corneal endothelial cells (CECs). This study investigated the use of a synthetic peptide hydrogel using poly-ε-lysine (pεK), cross-linked with octanedioic-acid as a potential substrate for CECs expansion and CE grafts. PεK hydrogel properties were optimised to produce a substrate which was thin, transparent, porous and robust. A human corneal endothelial cell line (HCEC-12) attached and grew on pεK hydrogels as confluent monolayers after 7 days, whereas primary porcine CECs (pCECs) detached from the pεK hydrogel. Pre-adsorption of collagen I, collagen IV and fibronectin to the pεK hydrogel increased pCEC adhesion at 24 h and confluent monolayers formed at 7 days. Minimal cell adhesion was observed with pre-adsorbed laminin, chondroitin sulphate or commercial FNC coating mix (fibronectin, collagen and albumin). Functionalisation of the pεK hydrogel with synthetic cell binding peptide H-Gly-Gly-Arg-Gly-Asp-Gly-Gly-OH (RGD) or α2β1 integrin recognition sequence H-Asp-Gly-Glu-Ala-OH (DGEA) resulted in enhanced pCEC adhesion with the RGD peptide only. pCECs grown in culture at 5 weeks on RGD pεK hydrogels showed zonula occludins 1 staining for tight junctions and expression of sodium-potassium adenosine triphosphase, suggesting a functional CE. These results demonstrate the pεK hydrogel can be tailored through covalent binding of RGD to provide a surface for CEC attachment and growth. Thus, providing a synthetic substrate with a therapeutic application for the expansion of allogenic CECs and replacement of damaged CE.

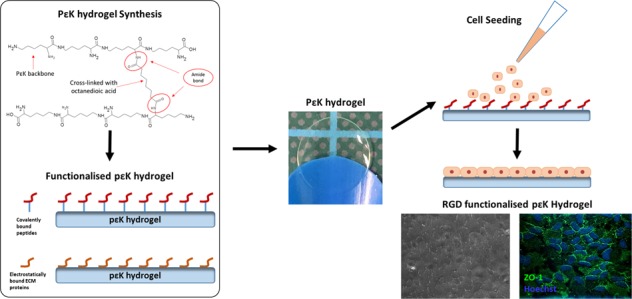

## Introduction

The corneal endothelium (CE) is the inner most layer of the cornea and is composed of a single monolayer of tightly packed, non-replicative endothelial cells on a thickened basement membrane (Descemet’s membrane (DM)). The primary role of the CE is to maintain the transparency of the cornea by regulating its hydration through a leaky barrier and active sodium-potassium adenosine triphosphase (Na^+^K^+^ATPase) pumps present on the membrane of corneal endothelial cells (CECs) [1, 2]. If CECs are lost, the remaining cells migrate and enlarge to ensure adequate cell coverage to maintain corneal transparency, however, there is a critical number of CECs required to maintain adequate pump function (>500 cells/mm^2^) [3]. Acute cell loss due to age, disease (such as Fuchs endothelial corneal dystrophy (FECD)), degenerative changes (bullous keratopathy) and other causes including infection, and physical or surgical trauma can eventually result in corneal oedema and decreased visual acuity [4].

Currently the only therapeutic treatment for corneal endothelial dysfunction is corneal transplantation using donor tissue. This treatment involves the replacement of the CE with donor CECs on their native DM, using most commonly, partial thickness grafts such as Descemet’s stripping automated endothelial keratoplasty (DSAEK) or Descemet’s membrane endothelial keratoplasty (DMEK). These procedures are not without complications as there is the risk of graft failure (due to rejection or gradual cell loss) [5, 6] and graft survival rate is only 70% at 5 years [7]. At present the ratio of donor tissue to recipient is 1:1 and there is a global shortage of corneas for transplantation, therefore, alternative therapeutic methods using expanded CECs are being developed as they offer the advantage of production of several endothelial grafts from one donor to treat multiple recipients [5, 8].

CECs possess limited replicative capacity but in vitro expansion is possible, while still maintaining phenotype and function [9, 10]. Currently, there are two potential modes of delivery of cultured CECs; direct cell injection into the anterior chamber or transplantation of an engineered graft comprising a cell monolayer on a carrier/scaffold [11–14]. Preclinical studies have shown conflicting functional outcomes using injected cells [11, 15–18], however, a clinical trial of 11 patients with bullous keratopathy did show injected CECs supplemented with Rho kinase (ROCK) inhibitor Y-27632 increased the density of CECs [19]. A recent publication directly comparing injected CECs with a tissue engineered graft of CECs in a rabbit model highlighted an important point [20]. When CECs were injected into the eye of a rabbit with DM removed, the CECs failed to improve corneal transparency or decrease corneal thickness and were later found to have failed to attach and form a monolayer. In FECD vision is adversely affected by deposition of focal excrescences, known as guttae, which are present in the central DM. The DM must be removed before delivery of an endothelial graft meaning injected cell therapy will not be suitable for these patients or late stage bullous keratopathy patients with DM scarring. In these cases there is clearly still a requirement for a tissue engineered graft that can also fulfil the role of the DM.

Previous studies have demonstrated that CECs can be expanded onto biological substrates, such as amniotic membrane [21], decellularised endothelium/stroma and lens capsule [22–25]. CECs grew as cell monolayers on these substrates and displayed a uniform shape and size, with cell to cell contacts and expressing typical CEC markers zonula occludens-1 (ZO1), Na^+^/K^+^ATPase and connexin 43. Substrates derived from natural polymers (collagen membranes [23, 24] and sponges [26], compressed collagen I [27, 28], silk fibroin [29] or synthetic polymers (poly(caprolactone)) or a combination of both (chitosan and poly(caprolactone) [30, 31]) have been used as materials to mimic the biological and mechanical properties of DM for culturing CECs. CECs proliferated on these substrates and maintained their typical morphology and phenotype. Natural polymers used as coating on culture dishes, such as collagen I [32] and collagen IV, fibronectin, gelatin, laminin and chondroitin sulphate have also been shown to be beneficial in promoting a cobblestone endothelial-like morphology [33–37].

The properties of synthetic substrates can be tightly controlled to produce a customised material with desired mechanical properties and surface functionalisation to increase e.g. cell adhesion. Synthetic hydrogels are an attractive option for CE replacement as they would allow the diffusion of water and biomolecules into the stroma. Our previous studies have shown that poly-ε-lysine (pεK) cross-linked with octanedioic-acid produces hydrogels that are transparent, are non-toxic to corneal epithelial cells in vitro and can be cast into contact lens moulds [38, 39]. In addition, following crosslinking of pεK hydrogels, free amine sites remain for either electrostatic or covalent binding of drugs, proteins or peptides to the pεK hydrogel. The adaptability of this synthetic material means the pεK hydrogel properties, such as stiffness and amine functionality, can be tailored to facilitate production of a stable endothelial monolayer.

The current study aimed to develop the synthetic pεK hydrogel as a substrate for the expansion of CECs. The objectives were to manufacture a thin pεK hydrogel film with optimum transparency and mechanical properties, which maintained sufficient free amines to allow binding of biomolecules (such as extracellular matrix (ECM) proteins and synthetic cell binding peptides (H-Gly-Gly-Arg-Gly-Asp-Gly-Gly-OH (RGD) and H-Asp-Gly-Glu-Ala-OH (DGEA)), to enable production of a confluent endothelial cell monolayer that maintained the characteristic CEC phenotype. We investigated the attachment and growth of both a human corneal endothelial cell line (HCEC-12) and primary porcine endothelial cells (pCEC) onto these pεK hydrogels and the effects of protein and peptide functionalisation of the pεK hydrogels. The results demonstrate, for the first time, that pεK hydrogels tailored to promote cell attachment can be used for the expansion of CECs, which displayed a characteristic phenotype in long term culture.

## Materials and methods

### PεK hydrogel synthesis and material testing

The pεK hydrogel was synthesised from pεK (Bainafo- Zhengzhou Bainafo Bioengineering Co. Ltd) cross-linked to 60% with octanedioic-acid (Sigma Aldrich, Dorset, UK) to a polymer density of 0.066 g/ml using N-hydroxysulfosuccinimide (NHS, CarboSynth ltd, Berkshire, UK) and 1-ethyl-3-(3-dimethylaminopropyl) carbodiimide (EDCI, CarboSynth ltd, Berkshire, UK). Specifically, pεK (0.692 g) was dissolved with dH_2_O (2 ml) in a 15 ml polypropylene universal (Starlab Ltd., Blakelands, UK). Octanedioic acid (0.210 g) was dissolved in dH_2_O (1 ml) with N-Methylmorpholine (NMM, Sigma, UK; 0.442 ml). This was placed on a roller until dissolved and added to the pεK solution. An aliquot of 5% Tween 20 (0.1 ml) was added. NHS (0.232 g) was dissolved in dH_2_O (1 ml). EDCI (1.157 g) was separately dissolved in distilled H_2_O (dH_2_O) (2 ml). The EDCI and NHS were mixed together and immediately added to the pεK/octanedioic acid solution before topping up the solution to a final volume (10 ml) with dH_2_O. This was inverted ten times. An aliquot of the polymer solution (40 μl) was pipetted into the condensation rings on the lid of a 48 well plate (Greiner Bio-One GmbH, Kremsmunster, Austria). These were incubated overnight at room temperature until the pεK hydrogel had polymerised, followed by 5 × 5 min dH_2_O washes.

Cast pεK hydrogel thickness was measured in the wet state using a modified Draper micromere gauge, the thickness of a sample of pεK hydrogels used for cell culture (4 for each repeat) were tested and they had a thickness range of 100–130 µm and a diameter of 8 mm. For material testing the pεK hydrogel solution was cast into a 90 mm petri dish. To analyse the surface hydrophobicity, contact angle measurements (DSA100, Krüss GmbH, Germany) were performed using a 5 µl drop size, 9 drops were dispensed randomly across the surface of the pεK hydrogel. The percentage water content of 1 mm diameter pεK hydrogels was measured. Samples were measured in the wet state and then left in a desiccator overnight to measure dry weight. Mechanical testing was performed until failure using dog bone punched samples with a width of 2.8 mm and gauge length of 10 mm, using the TST350 tensile tester (Linkam, UK) with a 20 N load cell at 100 µm/s strain rate. The thickness of the pεK hydrogel sheet was measured prior to punching, thickness values were inputted into the Linkam tensile tester to calculate the stress. For transparency testing a 6 mm punch was used. Samples were placed in a 96 well plate (Griener BioOne) and optical density was recorded across several wavelengths of the visible spectrum (460, 544, 560, 570 and 600 nm) using a FLUOstar spectrophotometer (BMG Labtech, UK), a Contaflex 75 contact lens was used as a control (Menicon, UK). All material testing was repeated with 3 synthesised pεK hydrogels to give an *n* = 3. The microstructure of the pεK hydrogel was investigated using Multimode 8 atomic force microscope (AFM) with Nanoscope V controller (Bruker, UK). A silicon nitride cantilever of spring constant 0.4 N/m was used in scanasyst mode. A 2 µm^2^ region was scanned at a rate of 1 Hz with 512 samples/line. The scans were subjected to 0^th^ order plane fit, to remove image bow.

### Functionalisation of pεK hydrogels

The pεK hydrogel was functionalised either by electrostatically binding ECM proteins onto the pεK hydrogel or via covalently binding peptides to the pεK hydrogel. All pεK hydrogels were washed in 70% (v/v) ethanol for 1 h (hr) and then washed extensively in sterile Phosphate Buffered Saline (PBS, Oxoid, UK).

The following proteins were electrostatically bound to pεK hydrogels (all Sigma unless stated otherwise): human fibronectin (50 µg/ml), rat tail collagen I (100 µg/ml), collagen IV from human placenta (100 µg/ml), chondroitin sulphate (20 mg/ml), laminin from human fibroblasts (50 µg/ml) and the commercial FNC coating mix ® (composed of fibronectin, collagen and albumin) (Enzo Lifesciences) for 1 h at 37 °C, followed by the removal of excess solution [40].

Prior to covalent binding, precast pεK hydrogels were washed 3 times with 10% NMM in dH_2_O solution then washed 3 times in dH_2_O. Cell binding peptide H-Gly-Gly-Arg-Gly-Asp-Gly-Gly-OH (RGD, Spheritech ltd, UK), and α2β1 integrin recognition sequence H-Asp-Gly-Glu-Ala-OH (DGEA, Sigma, UK) were covalently bound to precast pεK hydrogels via the available amine sites in a 0.25 M solution of NHS and EDCI dissolved in dH_2_0. RGD was bound at 100% loading capacity to available amine groups whereas DGEA was bound at 5% and 1% loading capacity. Specifically 127 mg of RGD was added to 0.072 g NHS and 0.12 g EDCI in 2.5 ml dH2O and mixed until dissolved. The precast pεK hydrogels were placed in a 24 well plate and 125 µl of the peptide solution was added to each well for 1 ½ h at RT on a rocker. For 5% and 1% DGEA either 0.656 or 0.131 mg, respectively, was added to 0.029 g NHS and 0.048 g EDCI in 1 ml dH2O and mixed until dissolved. The precast pεK hydrogels were placed in a 12 well plate and 250 µl of the peptide solution was added to each well for 1 ½ h at RT on a rocker. Peptide solution was then removed and 5 × 5 min dH_2_O washes were performed prior to washing in 70% ethanol.

### HCEC-12 cell culture

Immortalised, transformed human corneal endothelial cells HCEC-12 cells (HCEC-12 (RRID:CVCL_2064) ACC 646, DSMZ) were grown in culture medium Hams F12 and Medium 199 (1:1) (Invitrogen, UK) with 5% (v/v) fetal bovine serum (FBS) (Biosera, Labtech, UK). HCEC-12 cells were grown to 70–80% confluence and passaged using TrypLE Express (Invitrogen, UK) for 5 min. Detached cells were collected in culture medium and centrifuged at 1000 rpm for 5 min. Cell pellets were re-suspended in culture media and diluted to required cell density for use in experiments. HCEC-12 cells were used between passage 8–15 for experiments.

### Porcine corneal endothelial cell isolation and expansion

Fresh porcine eyes were obtained from 6-month old pigs within 6 h of slaughter from a local abattoir. The extra-ocular tissue was removed from eyes followed by a 2 min wash in PBS, (containing 1% (v/v) penicillin/streptomycin and amphotericin B (P/S/AmpB) (Sigma, UK)). Eyes were immersed in povidone iodine (3% (v/v) Medisave, UK) diluted in PBS for 2 min and were washed twice in PBS to remove excess povidone iodine. Isolation of CECs was performed as previously reported with minor modifications [37, 41]. Briefly, corneas were excised from whole globes making an incision 3–4 mm from the posterior limbus and the anterior segment was obtained making a circumferential cut along the incision. The lens, iris and ciliary body and trabecular meshwork tissue were carefully removed. The corneas were rinsed 2–3 times in fresh PBS containing 1% (v/v) P/S/AmpB to remove any unwanted cells. Corneas were placed endothelium side up in a sterile Bijou cap in a 12 well plate and incubated with 300 µl TrypLE Express (Invitrogen, UK) for 15 min at 37 °C, 5% CO_2_. Following incubation, CECs were isolated from both the central and peripheral regions as a single culture. CECs were released into the TrypLE Express solution by gentle scraping using a small inoculation loop to dislodge cells and pipetted into a well of a 6 well plate. A further 300 µl media was added to CE to ensure the capture of all endothelial cells. Isolated cells were re-suspended in culture medium (5 ml DMEM (Sigma, UK) with 10% FBS, 1% (v/v) P/S/AmpB)) and cultured in 6 well plates for 2 weeks and cell media was replaced every 2–3 days until confluence was achieved. Confluent cultures of porcine CECs showing characteristic regular hexagonal morphology were apparent after 2 weeks and were passaged into T25 flasks (passage 2) for further expansion and use for experimental studies at passage 3.

### Cell seeding onto pεK hydrogel

HCEC-12 cells and porcine CECs were dissociated from the culture dish using TrypLE Express for 10 min at 37 °C, 5% CO_2._ Following enzyme dissociation, CECs were re-suspended in 5 ml of media and centrifuged at 1000 rpm for 5 min. PεK hydrogels were transferred into 12 well tissue culture polystyrene (TCPS) plates and excess PBS was allowed to evaporate for approximately 15 min under sterile conditions. CECs were counted and seeded onto 8.5 mm diameter pεK hydrogels at a density of 1800 cells/mm^2^ (100 000 cells per pεK hydrogel) in a concentrated cell solution (50 µl) aliquoted to the centre of the pεK hydrogel and transferred to the incubator for approximately 2 h at 37 °C 5% CO_2_ to allow cell adhesion. After 2 h a further 2 ml of media was gently added to the well and the pεK hydrogels were incubated for 24 h at 37 °C 5% CO_2_. After 24 h in culture the pεK hydrogels plus attached cells were transferred into fresh 12 well TCPS plates with fresh pCEC culture media, thus excluding any pCECs that may have adhered to the bottom of the original 12 well TCPS plates. Cell medium was replaced every 2–3 days until confluence was achieved.

### Analysis of cell adherence to pεK hydrogel

The number of cells adhered to the pεK hydrogel surface was measured as a longitudinal study at 24 h, 4 days, 7 days, and 5 weeks, replacing the media every 3 days for long term cultures. Cells which remained rounded were not deemed adhered to the pεK hydrogel. To quantify the number of cells adhered to the pεK hydrogel, 3–5 randomised fields of view were imaged per pεK hydrogel, using phase contrast microscopy on live cells using a Nikon Eclipse Ti-E microscope (Nikon, UK). The same pεK hydrogel with cells was imaged at the specified time points until the experiment was terminated at the final time point. Cells were quantified using ImageJ 1.48 v, images were processed to enhance brightness and contrast using the threshold tool to discriminate cell boundaries. The plugin Cell Counter tool on Image J was used to mark and count individual cells.

### Immunocytochemistry

PεK hydrogel/cell constructs were washed briefly in PBS and fixed in 10% (v/v) neutral buffered formalin (Sigma, UK) for 10 min. HCEC-12 and pCECs were permeabilised with 0.1% (v/v) Triton X 100 (Sigma, UK) in PBS) at RT for 5 min. Non-specific binding sites were blocked for 30 min at RT with 300 µl blocking buffer (10% (v/v) Normal Goat Serum (Sigma, UK) in PBS). Samples were incubated with primary antibody zonula occludens 1 (ZO-1) (Invitrogen Rabbit polyclonal 40–2200) 250 μg/ml, 1:40) and Na^+^/K^+^ATPase (Santa Cruz #sc-58628, Mouse monoclonal IgG_1_, 200 µg/ml, 1:20) overnight at 4 °C then washed 3 times for 5 min with PBS (0.05% (v/v) Tween-20 (Sigma, UK)). PεK hydrogels were incubated with secondary antibody (Alexa Fluor 488 Goat anti-Rabbit and Alexa Fluor 488 Goat anti-Mouse (Invitrogen, UK)) and isotype control for 1 h in the dark at RT, followed by 3 PBS washes for 5 min each. PεK hydrogels were stained with Hoechst 33442 (Thermo Fisher, UK) for 15 min, washed in PBS and imaged using Nikon Ti microscope, using the Z stack option to create a compiled image for brightfield images. A Zeiss confocal LSM800 and Nikon Eclipse Ti-E microscope was used for immunofluorescence images using z stacks to compile a focused image.

### Statistical analyses

Data was presented as the mean and standard deviation (SD) of at least three independent experiments (*n* = 3), with each independent experiment being performed on a minimum of 3 pεK hydrogels for each condition. One-way analyses of variance (ANOVA) and post-hoc Tukey’s HSD test were performed to evaluate statistical significances between groups of samples. All statistical analyses were performed using Minitab 17.0. Statistical significance is shown using the *p*-value of *p* < 0.05 (*).

## Results

### PεK hydrogels are transparent and easy to manipulate

**Fig. 1:**
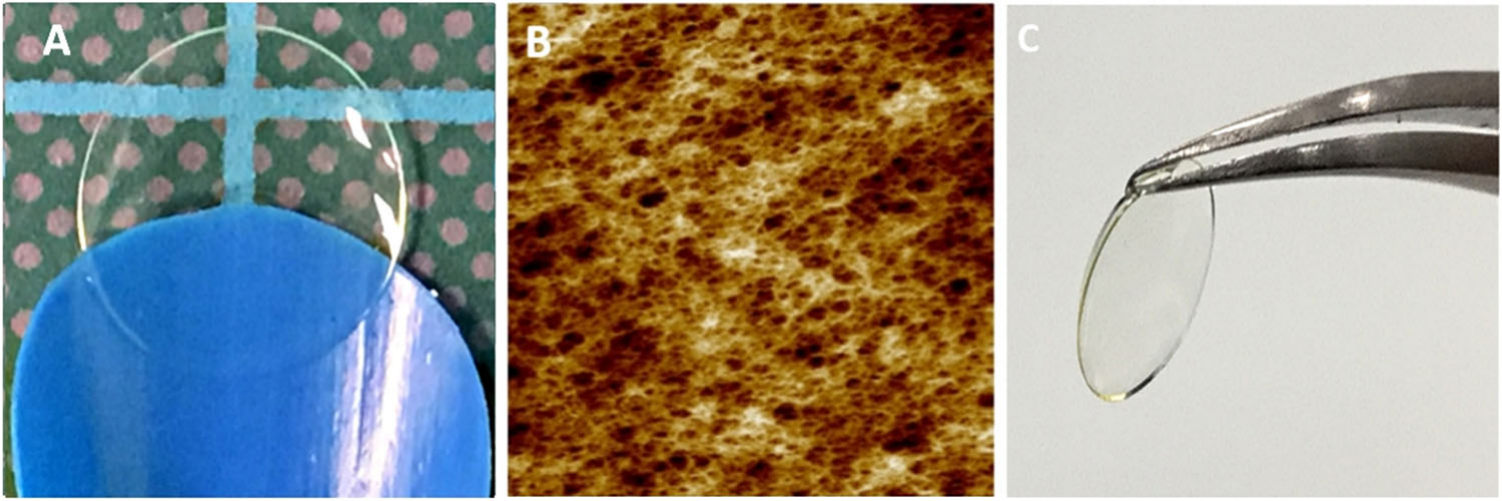
**a** Representative photograph of a pεK hydrogel cross-linked to 60% with octanedioic-acid to a polymer density of 0.066 g/ml demonstrating a thin transparent hydrogel. **b** Atomic force micrograph detailing the microporous structure of the same pεK hydrogel. **c** Image to show how the hydrogels can be manipulated easily using forceps

Thin, transparent pεK hydrogel films (Fig. 1a) were fabricated with an open porous structure demonstrated by AFM (Fig. 1b), an average of 118 µm (SD 16 µm) thickness was observed for gels synthesised for cell culture (Table 1). The pεK hydrogels had an average stiffness and ultimate tensile strength of 0.11 and 0.04 MPa, respectively (Table 1). As expected, contact angle analysis revealed the pεK hydrogel was hydrophilic with an average contact angle of 18.1° (SD 1.26) along with a high water content of 91.25% (SD 1.65) (Table 1). The pεK hydrogels had excellent transparent properties across all wavelengths of the visible spectrum, for example a 99.08% (SD 0.57) light transmission at 560 nm wavelength was observed (Table 1) (560 nm was chosen as a representative wavelength as values did not differ across the various wavelengths). This was not significantly different to the contact lens positive control, 100.4% (SD 1.28) (*p* > 0.05). The mechanical properties of the pεK hydrogels meant that they were robust enough to be manipulated with forceps and handled as they would be in a surgical procedure (Fig. 1c).

**Table 1:** Physical properties of a pεK hydrogel cross-linked to 60% with octanedioic-acid to a polymer density of 0.066 g/ml *n* = 3

60% cross-linked octanedioic-acid with 0.066 g/ml pεK	Average	Standard deviation ±
Thin film thickness (µm)	118	16
Young’s modulus (MPa)	0.11	0.01
Ultimate tensile strength (MPa)	0.04	0.004
Contact angle (deg)	18.1	1.26
% Water content	91.25	1.65
% Transparency (560 nm)	99.08	0.57

### HCEC-12 cell line, but not pCECs, adhered and remained attached on pεK hydrogels after 7 days

**Fig. 2:**
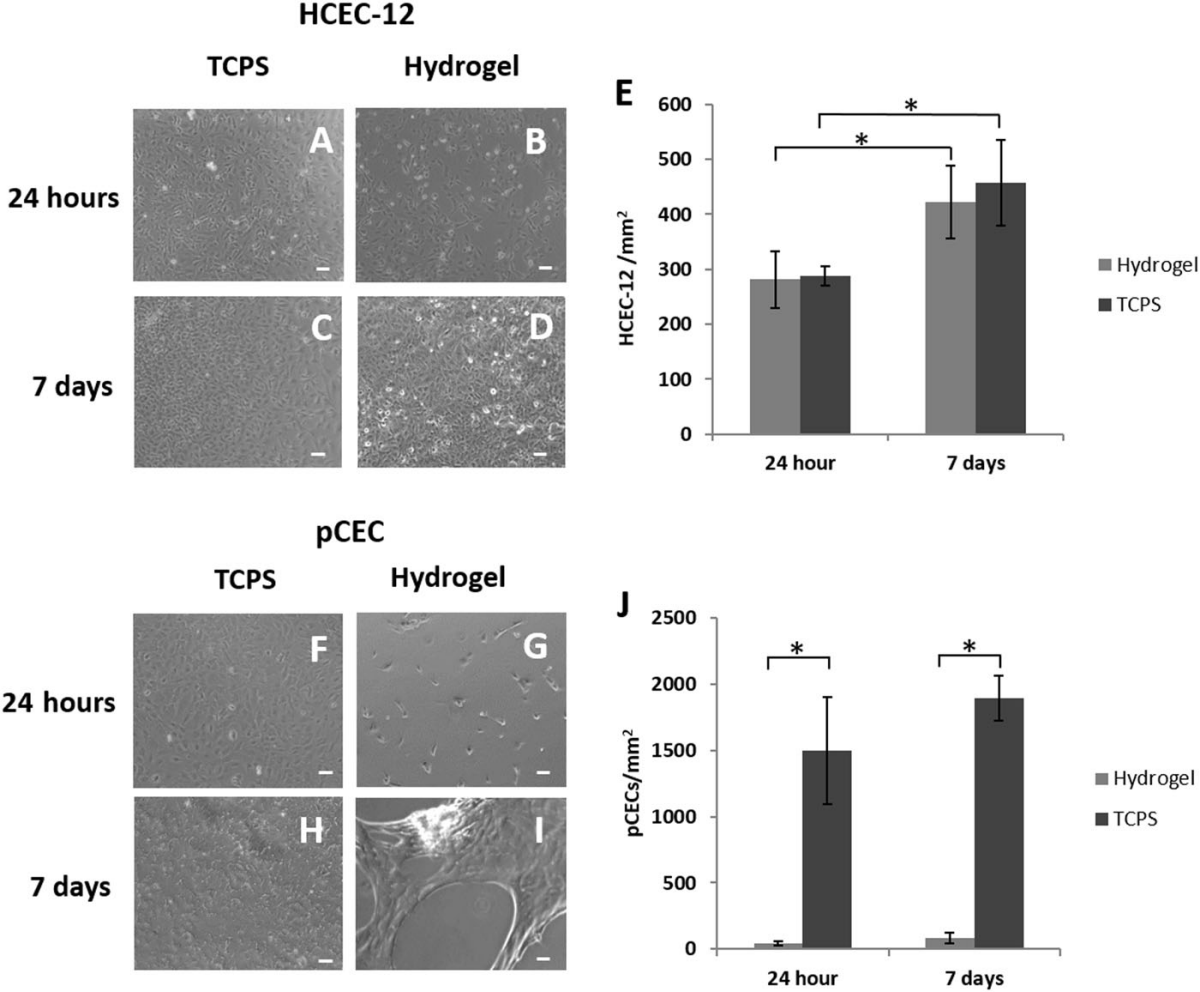
HCEC-12 and pCEC expansion on a pεK hydrogel cross-linked to 60% with octanedioic-acid to a polymer density of 0.066 g/ml. HCEC-12 cells and pCECs were seeded onto pεK hydrogels at a density of 1 × 10^5^ cells per pεK hydrogel (1800 cells/mm^2^). Representative phase contrast images of HCEC-12 cells expanded 24 h on **a** TCPS and **b** pεK hydrogel or at 7 days and on **c** TCPS and **d** pεK hydrogel. **e** Quantification of cell density (cells/mm^2^) at 24 h and 7 days post seeding of HCEC-12 cells on hydrogels. Representative images of pCECs seeded at 24 h on **f** TCPS and **g** pεK hydrogel or at 7 days on **h** TCPS and **i** pεK hydrogel. **j** Quantification of cell density (cells/mm^2^) at 24 h and 7 days post seeding of pCECs on hydrogels. Scale bar 100 μm, *n* = 4 for HCEC-12 pεK hydrogels, *n* = 3 for pCECs. Error bars show SD. Symbol * represents significant difference (*p* < 0.05) using one way ANOVA and post-hoc Tukey’s HSD analysis across groups

The HCEC-12 cells adhered to the pεK hydrogel at 24 h post seeding with an average cell density of 281.50 cells/mm^2^ (SD 51.81), comparable to TCPS 288 cells/mm^2^ (SD 17.78) (no significant difference (*p* > 0.05)) (Fig. 2a, b). By 7 days HCEC-12 cells covered the pεK hydrogel surface to form a cell monolayer with an average cell density of 422.9 cells/mm^2^ (SD 87.88) (Fig. 2c, d), which was comparable to TCPS 457.13 cells/mm^2^ (SD 78.32) (*p* > 0.05) (Fig. 2e). The number of HCEC-12 cells covering the pεK hydrogel surface at 7 days was significantly higher than at 24 h post seeding (Fig. 2e) (*p* < 0.05).

Primary pCEC adherence on the pεK hydrogel was lower when compared to HCEC-12 cells or pCECs on TCPS at 24 h with an average cell density on the pεK hydrogel of 43.86 cells/mm^2^ (SD 13.23) (Fig. 2f, g, j), compared to TCPS 1495.64 cells/mm^2^ (SD 403.12), significantly different (*p* < 0.05). At 7 days pCECs covered the surface of the pεK hydrogel to achieve an average cell density of 81.82 cells/mm^2^ (SD 38.49) (Fig. 2h, i, j), significantly different compared to TCPS 1893.27 cells/mm^2^ (SD 170.41) (*p* < 0.05). Although there were regions of high pCEC density on the pεK hydrogels, it was evident that pCECs had detached from regions of the pεK hydrogel surface as sheets at 7 days (Fig. 2i).

### Pre-adsorbing collagen I, collagen IV and fibronectin onto pεK hydrogels improved the adhesion and morphology of pCECs

**Fig. 3:**
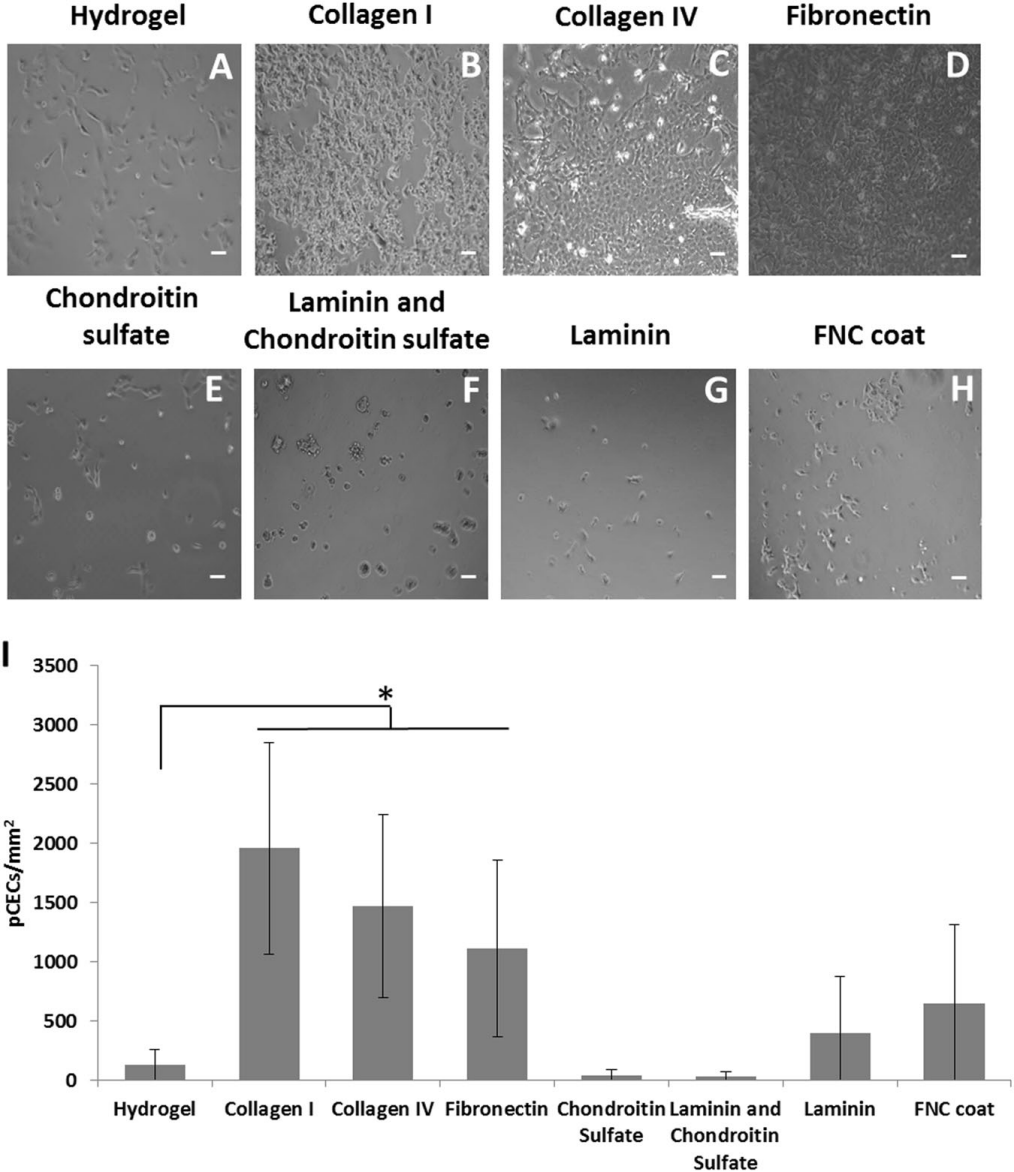
Representative phase contrast images of adhesion of pCECs on a pεK hydrogel cross-linked to 60% with octanedioic-acid to a polymer density of 0.066 g/ml pre adsorbed with ECM proteins. PεK hydrogels were uncoated **a** or electrostatically bound for 1 h with proteins **b** collagen I 100 μg/ml, **c** collagen IV 50 μg/ml, **d** fibronectin 50 μg/ml, **e** chondroitin sulfate 20 mg/ml, **f** Laminin and chondroitin sulfate, **g** laminin 50 μg/ml, and **h** FNC coat. pCECs were seeded onto hydrogels at a cell density of 1800 cells/mm^2^. PεK hydrogels were imaged 24 h post seeding. Scale bar 100 μm, *n* = 3. I Quantification of cell density (cells/mm^2^). Symbol * denotes significantly different *p* < 0.05 compared to pεK hydrogel only using ANOVA and post-hoc Tukey’s HSD analysis, Error bars show SD

There was increase in the number of pCECs adhered to the pεK hydrogel surface which were pre adsorbed with either collagen I, collagen IV or fibronectin at 24 h, compared to the untreated pεK hydrogels (Fig. 3a–h). An average cell density of 1958 cells/mm^2^ (SD 896.31) for collagen I, 1471.6 cells/mm^2^ (SD 774.43) collagen IV and 1112.19 cells/mm^2^ (SD 744.55) for fibronectin was achieved compared to 128.43 cells/mm^2^ (SD 129.07) for untreated pεK hydrogels (*p* < 0.05) (Fig. 3i). There was no significant difference between the numbers of pCECs on the pεK hydrogel surface whether it had been pre-adsorbed with collagen I, IV or fibronectin (Fig. 3i) (*p* > 0.05). There was variation in the cell number across the pεK hydrogel surface, thus accounting for the large standard deviations at the 24 h time point. The addition of FNC coat, chondroitin sulfate, laminin or a mix of both chondroitin sulfate and laminin did not significantly improve the cell density on the pεK hydrogel (*p* > 0.05), compared to the untreated pεK hydrogel (Fig. 3a, e–i).

**Fig. 4:**
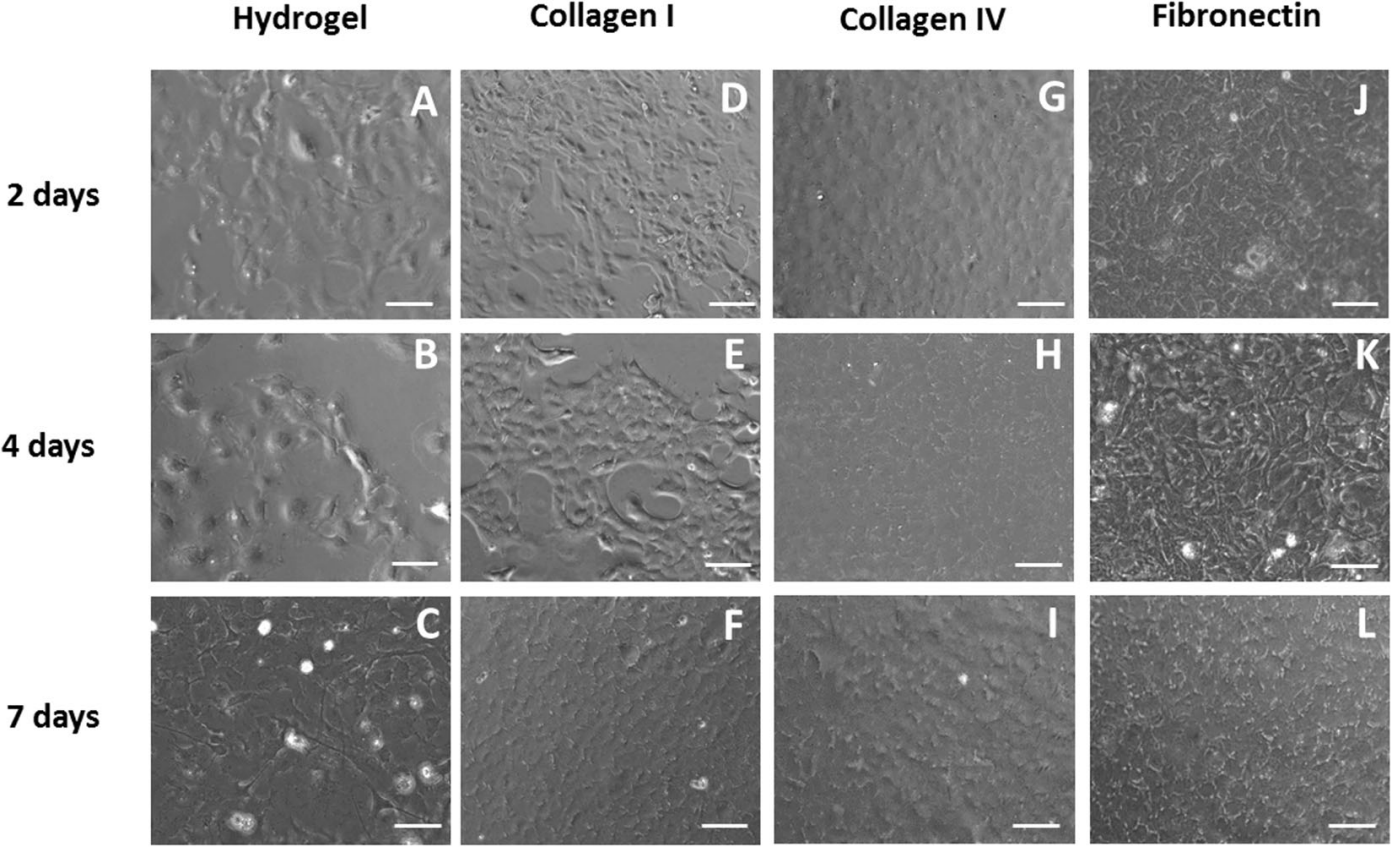
Adhesion and expansion of pCECs on a pεK hydrogel cross-linked to 60% with octanedioic-acid to a polymer density of 0.066 g/ml pre-adsorbed with ECM proteins. PεK hydrogels (**a**–**c**) were electrostatically bound with proteins for 1 h with **d**–**f** collagen I 100 μg/ml, **g**–**i** collagen IV 50 μg/ml and **j**–**l** fibronectin 50 μg/ml. pCECs were seeded onto pεK hydrogels at a cell density of 1800 cells/mm^2^. PεK hydrogels were imaged 2 days, 4 days and 7 days post seeding. Scale bar 100 μm

Representative images of pCECs on the pεK hydrogels with pre-adsorbed collagen I, collagen IV and fibronectin at 2 days, 4 days and 7 days, showed that pCECs formed confluent monolayers at 7 days across the pεK hydrogel surfaces (Fig. 4a–l). The pCEC monolayers remained attached to the pεK hydrogel surface at 7 days on pεK hydrogels pre-adsorbed with collagen I, collagen IV and fibronectin, in comparison to the untreated pεK hydrogel, which displayed regions of pCECs detachment (Fig. 4c, f, i, l). Representative images of pCECs show compact monolayers exhibiting the characteristic hexagonal appearance typical of primary pCECs on these surfaces, in comparison to the untreated pεK hydrogel.

### RGD but not DGEA functionalisation of pεK hydrogels increased adhesion of pCECs in long term cultures

**Fig. 5:**
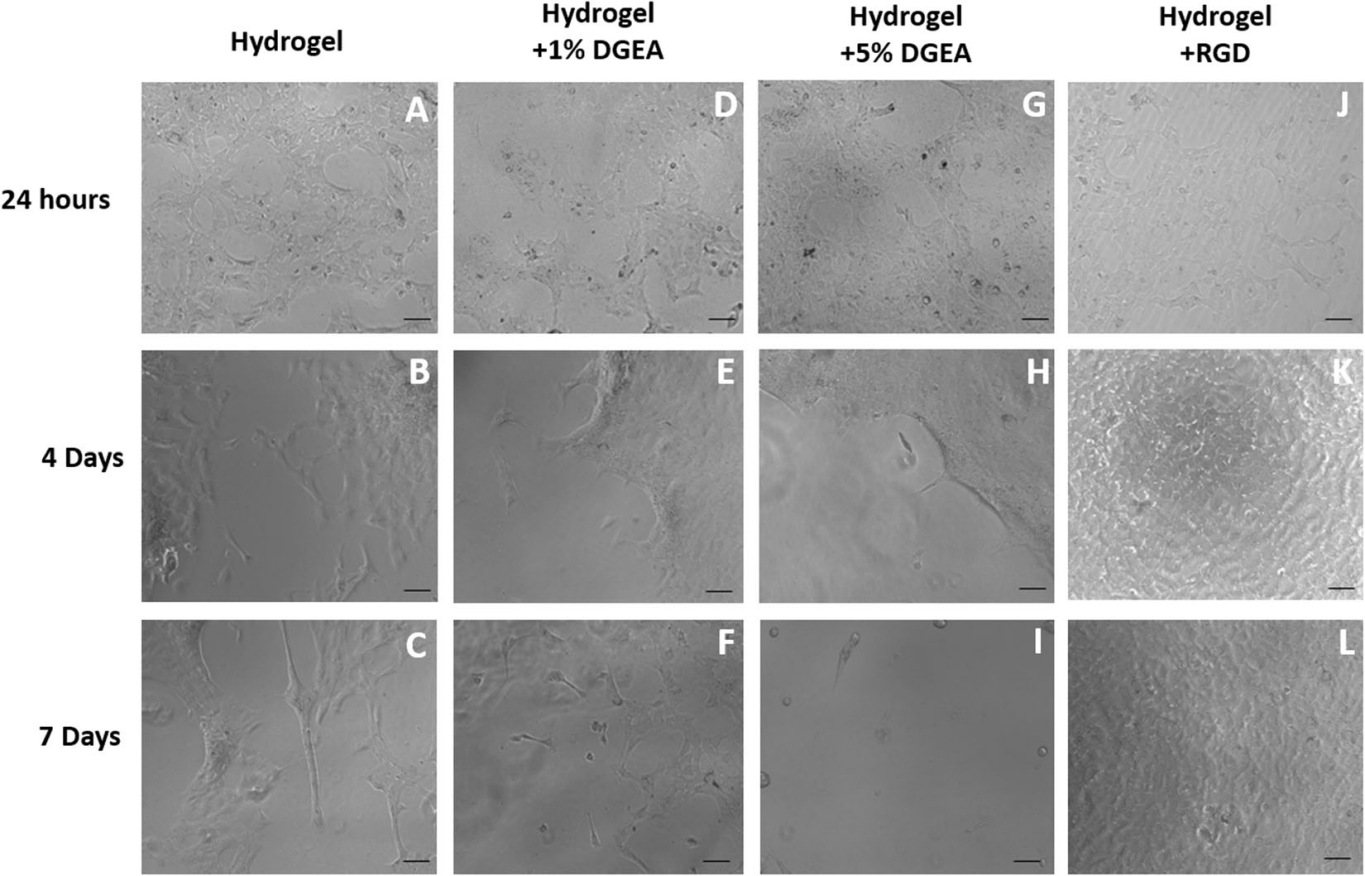
Primary pCECs seeded on pεK hydrogels cross-linked to 60% with octanedioic-acid to a polymer density of 0.066 g/ml and non-functionalised or functionalised with DGEA and RGD. Representative phase contrast images of pCECs expanded for 24 h, 4 and 7 days respectively on **a**–**c** non-functionalised pεK hydrogels, **d**–**f** DGEA 1% functionalised pεK hydrogels, **g**–**i** DGEA 5% functionalised pεK hydrogels and **j**–**l** RGD functionalised pεK hydrogels. Scale bar 100 μm

It was demonstrated by phase contrast microscopy that there were less pCEC coverage on the non functionalised pεK hydrogels (Fig. 5a–c), DGEA 1% (Fig. 5d–f), DGEA 5% (Fig. 5g–i) at 24 h, 4 days and 7 days, when compared to the RGD (Fig. 5j–l) pεK hydrogels. Cell adhesion to the DGEA functionalised pεK hydrogels was low and comparable to the non-functionalised pεK hydrogel.

**Fig. 6:**
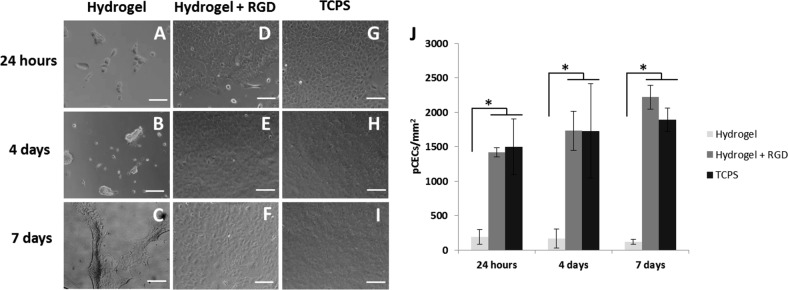
Primary pCECs seeded onto a pεK hydrogel cross-linked to 60% with octanedioic-acid to a polymer density of 0.066 g/ml with/without RGD functionalisation. PεK hydrogels were imaged at 24 h, 4 days and 7 days. Representative images of cells expanded on **a**–**c** non-functionalised or **d**–**f** RGD functionalised pεK hydrogels or **g**–**i** TCPS. **j** Quantification of cell density (mm^2^) on pεK hydrogels at specified time points. pCECs were seeded onto pεK hydrogels at a cell density of 1800 cells/mm^2^_._ Scale bar 100 µm, *n* = 3. Error bars show SD. Symbol * represents significant difference (*p* < 0.05) using one way ANOVA and post-hoc Tukey’s HSD analysis across groups

The number of pCECs adhered to the RGD functionalised pεK hydrogel surface was monitored by phase contrast imaging of live pCECs on pεK hydrogels at 24 h, 4 days and 7 days, compared to non functionalised pεK hydrogels and TCPS (Fig. 6a–i). The average number of cells covering the RGD functionalised pεK hydrogels surface at 24 h was 1418 cells/mm^2^ (SD 65.07) and was comparable to TCPS with an average of 1495 cells/mm^2^ (SD 403.12) (Fig. 6j). No significant difference was observed between the RGD functionalised pεK hydrogel and TCPS (*p* > 0.05), at any time point (Fig. 6j). The number of pCECs covering the non functionalised pεK hydrogels surface at 24 h was 187 cells/mm^2^ (SD 104.99).

By day 4 and 7 the number of pCECs on the RGD functionalised pεK hydrogel remained comparable to TCPS (Fig. 6e, f, h, i). On day 4, 1729.12 cells/mm^2^ (SD 279.95) covered the RGD functionalised pεK hydrogel surface, comparable to TCPS 1728.29 cells/mm^2^ (SD 685.54) (*p* > 0.05) (Fig. 6j). On day 7, 2219.19 cells/mm^2^ (SD 171.09) covered the RGD functionalised pεK hydrogel surface, comparable to TCPS 1893.27 cells/mm^2^ (SD 170.41) (*p* > 0.05) (Fig. 6j). The pCECs on the non-functionalised pεK hydrogel had detached from the surface by 7 days (Fig. 6c).

**Fig. 7:**
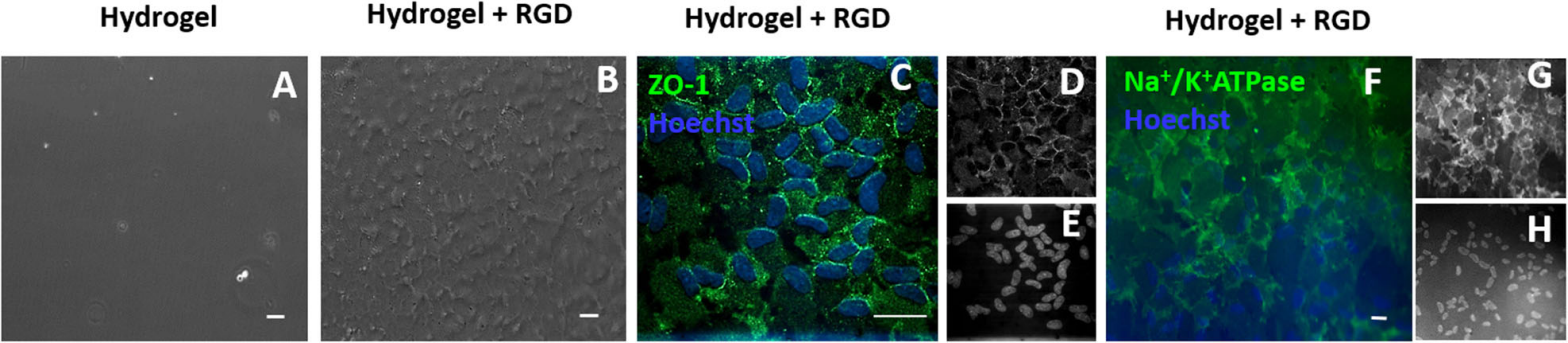
Long term culture of pCECs expanded on hydrogels. **a** Non-functionalised hydrogel. **b** RGD functionalised hydrogel 5 weeks post seeding of pCECs. Scale bars 100 µm. **c** ICC of ZO-1 (green) and Hoechst 33342 (blue). Monocoloured images: **d** ZO1 and **e** Hoechst 33342. **f** Na^+^/K^+^ATPase (green) and Hoechst 33342 (blue) expression. Monocolour images **g** Na^+^/K^+^ATPase and **h** Hoechst 33342. Scale bars 20 µm

pCECs on RGD functionalised pεK hydrogels were maintained in culture for up to 5 weeks post seeding and a monolayer of cells remained adhered to the pεK hydrogel surface without any regions of cell detachment, compared to the non-functionalised pεK hydrogel where no cells were present on the surface (Fig. 7a, b). The cell density on these pεK hydrogels at 5 weeks post culture (longest time point monitored) was an average of 2548 cells/mm^2^ (SD 159.48). pCEC monolayers displayed characteristic CEC hexagonal morphology and immunofluorescence staining for ZO-1 was localised at the cell-cell junctions, indicating tight junction formation (Fig. 7c–e) To validate the presence of a functional monolayer, immunocytochemistry for Na^+^/K^+^ATPase showed positive membrane staining of pCECs, suggesting the presence of a functional Na^+^/K^+^ATPase pump (Fig. 7f–h).

## Discussion

Corneal endothelial injury or dysfunction is currently treated with a corneal transplant with one donor endothelium being used to treat one patient. The opportunity to expand CECs on tissue engineered substrates offers an important alternative treatment to enable multiple recipients to be treated from a single donor. We have demonstrated in this study that we were able to fabricate thin, transparent pεK hydrogels, which can be fine-tuned to control for cell adhesion, enabling the in vitro expansion and support of primary CECs whilst maintaining their phenotypic markers. It was determined that pre-adsorption of proteins collagen I, collagen IV and fibronectin increased pCEC adhesion on pεK hydrogels. Functionalisation of the pεK hydrogel surface with the RGD peptide increased cell adhesion and expansion of pCECs and at 5 weeks these cells had formed a monolayer with expression of ZO-1 and Na^+^/K^+^ATPase, indicating the formation of tight junctions and presence of functional pumps, respectively.

PεK hydrogels used in this study are synthesised from pεK and dicarboxylic acids using carbodiimide chemistry and can be easily and rapidly manufactured from relatively inexpensive reagents, however, we appreciate that there is some variability in our current method of the pεK hydrogel manufacturing process, and parameters such as temperature and humidity would need to be more tightly controlled to upscale the process to a more reproducible method for commercial use. A significant advantage of pεK hydrogels is that we can tailor the mechanical properties of the pεK hydrogel to fit specific requirements. In this study, a pεK hydrogel with polymer density 0.066 g/ml cross-linked to 60% with octanedioic-acid was initially used as previous research had demonstrated that this pεK hydrogel was robust enough to handle easily [38, 39]. The pεK hydrogels had a modulus of 0.11 MPa and ultimate tensile strength of 0.04 MPa. These figures differ from the modulus of human DM, the substrate for the native endothelium, which is 0.05 MPa (range 0.02–0.08) and the ultimate tensile strength, which is 0.3 Mpa [42]. The important point to note, however, is that the hydrogel can be easily manipulated, as they would be during DSAEK surgery, without significant loss of cells or substrate integrity. Other materials that have been trialled as a substrate for an engineered endothelial layer such as pre-wet silk fibroin have demonstrated a Young’s modulus of 0.022 MPa and ultimate tensile strength of 2.1 MPa [43], however these materials have been too fragile to handle. We did not intend to produce a material with mechanical properties approximating the DM, as one of the problems with DMEK surgery is the high level of surgical skill required to handle the DMEK scroll [44]. It is a delicate tissue that can be easily damaged during the stripping and unfolding procedures so we aimed to produce a hydrogel that had improved robustness and was easier to unfold; effectively an intermediate between a DMEK and DSAEK graft combining the advantages of DSAEK tissue in terms of ease of handling and unfolding with the superior visual outcomes of the thinner DMEK tissue. In a recent review [45] the authors discuss the relative mechanical properties of natural materials for corneal tissue engineering and demonstrate that the elastic modulus varies considerably depending on the orientation and depth of the tissue sample (cornea and amniotic membrane) and the method of testing (uniaxial tension, compression or microindentation) but are generally in the range 0.3 to 3.0 MPa. In contrast to the natural tissues, the mechanical properties of purified materials such as collagen 1 and silk fibroin can be tailored by optimising the cross-linking. A similar approach can be taken with the pεK used in this study leading to greater control over the design of the substrate for both cell interactions and surgical handling. In comparison to collagen 1 and silk fibroin the pεK is inexpensive and readily available as a pharmaceutical grade material.

Substrates for the replacement of CE must be porous, thin and transparent, to allow the passage of fluid and nutrients from the aqueous humour into the stroma, as well as allowing the passage of light. We have demonstrated in this current study that our pεK hydrogel has an open porous network, observed via AFM, and that it is possible to cast thin pεK hydrogels (~100 µm). The pεK hydrogel was hydrophilic (contact angle 18.1°), most likely due to the presence of amines on the surface and the high water content within the pεK hydrogel (water content of 91.25%), had excellent transparency allowing the passage of light across all wavelengths of the visible spectrum (99.08% light transmission), and excellent cytocompatibility for other ocular applications. One reported example is the potential use of these pεK hydrogels as antimicrobial contact lenses, demonstrating that the pεK hydrogels showed good cytocompatibility with corneal epithelial cells [38].

The pεK hydrogel provides a platform that can be modified and tailored to specific cell requirements to control for cell attachment. As the crosslinking density of the pεK hydrogel increases, the number of free amine functional groups decreases and the stiffness of the pεK hydrogels increases. Both these properties can influence the interaction of cells with surfaces, thus altering cell activity and attachment. Our synthesised pεK hydrogel theoretically has 40% of the amine groups unbound and free. The amine surface of the pεK hydrogel not only promotes cell interactions but provides useful binding sites for biomolecules allowing specific functionalisation of the surface, either by pre-adsorption of ECM proteins or covalent binding of additional peptides. The non-functionalised pεK hydrogel seeded with the HCEC-12 cell line showed promising results, forming a confluent monolayer of cells with characteristic CEC hexagonal morphology and the numbers of cells present were comparable to cells on TCPS. However, primary pCEC attachment was significantly lower and although confluent monolayers were achieved, by 7 days, detachment of cell monolayers from the pεK hydrogel was observed. The differences between cell line and primary cells, with the primary pCECs detaching away from the pεK hydrogel surface as sheets, suggests the primary cells had a stronger attachment to one another rather than the material surface perhaps due to less deposition of a basement membrane (BM), although this would require confirmation by characterising the deposited ECM. The HCEC-12 cell line may have a lower requirement for cell adhesion or secrete more BM proteins in a short period of time compared to the pCECs to enable maintenance of a cell monolayer. We and others have reported that primary human corneal epithelial cells require long term culture (>3 weeks) to secrete substantial amounts of basement membrane proteins [46] and that the presence of neighbouring cells (stromal [46] or corneal endothelial cells [47]) in organotypic 3D cultures leads to the synthesis of a nearly continuous basement membrane. One mechanism that could explain this effect is that the neighbouring cells may produce a cytokine or matrix component that stimulates the epithelial cells to differentiate and assemble a basement membrane. It is likely that the stromal or epithelial cells also signal to the endothelial cells in a feedback loop. In the present study, primary endothelial cells were cultured in isolation so are not exposed to any such signalling, consequently, production of their own matrix may be substantially delayed. In order to ameliorate this, addition of exogenous ECM proteins on culture surfaces or biomaterials is a viable option. Modification of the pεK hydrogel with peptides/proteins clearly elicits different cellular responses, however, in this study we have not quantified the amount of bound peptide/proteins as it is challenging in this setting. The peptide structure of the pεK hydrogel masks the spectroscopic peaks of the attached peptides if using Fourier transform infrared spectroscopy (FTIR) and reagent absorption by the hydrogel creates considerable variability in background readings in ELISA based assays.

The DM is composed predominantly of collagen IV, collagen VIII, laminin and fibronectin [48]. Others have shown that mimicking the structure of DM, for example, with a dense layer of collagen IV and laminin (bovine cells) or collagen IV alone (human cells) encouraged endothelial cells to form high density monolayers that adhered to an underlying collagen I substrate [49, 50]. ECM proteins and substrates that mimic mechanical and biological properties of DM have been shown to be more favourable, increasing cell attachment and maintaining characteristic endothelial cell morphology [33, 51, 52]. In this present study we showed that electrostatic binding of ECM proteins (collagen I, collagen IV and fibronectin) to the surface of our pεK hydrogels improved attachment, maintenance of a cell monolayer and sustained the cell sheet adhesion at 7 days. Another study demonstrated that human CECs cultured on collagen I, collagen IV, fibronectin and commercial FNC coat attached well and spread but were only weakly attached to laminin and TCPS control plates [53]. This agrees with the findings of our study that the laminin did not increase adhesion of the primary porcine cells. Others have also modified the surface of their materials to increase cell attachment. Silk fibroin lacks ECM proteins and studies have shown that modification of the substrate via the incorporation of pre-adsorbed ECM proteins (collagen IV) onto the surface, promoted attachment and proliferation of human CECs [29]. Palchesko et al. also demonstrated that bovine CECs could be cultured on a poly(dimethylsiloxane) surface and the adhesion and morphology of CECs was improved with collagen IV proteins [40].

Although coating with natural ECM molecules appears to be beneficial to CEC adhesion and growth, they do have some disadvantages. An interesting solution to this problem was described by Rizwan et al. in 2017 using gelatin methacrylate (GelMA) as the base for a tissue engineered graft [54]. Instead of coating the surface of the hydrogel with ECM proteins themselves they nano-patterned the surface to mimic the nanoscale topographical cues contained on in vivo extracellular matrix. They showed that the topographical cues were able to improve the cell functions and phenotype of a CEC monolayer. It is preferable to use chemically and physically defined ECM mimics that can be reliably reproduced so this approach could be useful if direct functionalisation of the surface with synthetic peptides is not possible. We have successfully shown that we are able to functionalise the pεK hydrogel through covalent binding of synthetic ECM adhesive peptides, DGEA and RGD. Integrins form an important and widespread group of adhesion receptors as they interact with proteins of the extracellular matrix or with the receptors expressed on cells as shown in the binding of the leukocyte integrins to the intercellular adhesion molecules (ICAMs) [55]. DGEA (Asp-Gly-Glu-Ala), corresponding to residues 425–438 of the Type 1 collagen sequence, has been identified as a recognition motif used by the type I collagen to bind to α2β1 integrin [56]. The RGD peptide (Arg-Gly-Asp) is the most widely studied adhesive peptide and is the principal integrin binding domain present within ECM proteins such as fibronectin, vitronectin, fibrinogen and others. PεK hydrogels functionalised with RGD did facilitate pCEC adhesion, however, pCECs were unable to adhere to the surface of pεK hydrogels functionalised with DGEA. DGEA therefore did not appear to serve as an adhesion ligand for pCECs in this context. These results are consistent with a previously reported study describing rat calvarial osteoblasts and MC3TT3-E1 osteoblasts demonstrating limited adhesion on hydrogel surfaces presenting the DGEA peptide, which was described as ‘weakly adhesive’ [57, 58]. The glycine-phenylalanine-hydroxyproline-glycine-glutamate-arginine (GFOGER) peptide could be an alternative collagen I peptide to trial in the future. It known to provide a significant basal level of adhesivity along with RGD [59] and the integrin α2β1 recognizes the GFOGER motif in residues 502–507 of the α1 chain of type I collagen. Not only has the α2β1 integrin been identified on corneal endothelial cells [60] but the expression of β1 integrin has also been shown to be upregulated in cultured corneal endothelial cells when compared with native tissue, so this fact can be exploited with designing peptide functionalisation for tissue engineered endothelial grafts [61].

The biomimetic RGD domain is able to promote cell adherence to the pεK hydrogel surface and improve the cell/material interactions for many different applications [62, 63]. The advantage of using an RGD peptide instead of a natural protein is a reduction in immune reactivity or pathogen transfer, synthesis is easy and inexpensive and it can be performed in a controlled and defined manner [64]. For example, the coupling of the RGD peptide to silk was beneficial in enhancing the cell attachment and proliferation of corneal stromal cells in tissue engineering of the corneal stromal lamellae, which when implanted into an animal model, maintained their functionality [65, 66].

This study has demonstrated that pεK hydrogels offer a compatible substrate to support the expansion and growth of a human corneal endothelial cell line and primary pCECs in vitro. We found that the addition of pre-adsorbed collagen I, collagen IV and fibronectin increased the cell adhesion onto the pεK hydrogel and the cells remained attached at 7 days. The free amine sites on the pεK hydrogel enabled the functionalisation of the pεK hydrogel surfaces with RGD and DGEA cell binding peptides. The results demonstrated that bound RGD increased cell adhesion compared to non-functionalised pεK hydrogels and maintained cell attachment up to 5 weeks in culture. Long term cultures exhibited a characteristic CEC phenotype which showed the formation of tight junctions at the cell boundary and expression of Na^+^/K^+^ATPase demonstrating the functionality of the cell monolayer.

## Conclusions

Tissue engineered corneal endothelial grafts are being developed by a number of research groups in order to alleviate pressure on the demand for transplant tissue due to a worldwide donor cornea shortage. Our pεK hydrogel has great potential as a clinically viable option as the peptide is readily available in a purified state, its mechanical properties are customisable by design of the cross-linking density and it can be surface functionalised via free amine groups for any particular application. We have tailored the mechanical properties to enable easy handling of the graft by surgeons and functionalised the surface to improve corneal endothelial cell attachment. Successful development along the translational pipeline could see this technology being delivered to patients to treat endothelial dysfunction.
